# Supplementary material for: The Antitumor Potential of Sicilian Grape Pomace Extract: A Balance between ROS-Mediated Autophagy and Apoptosis
Source: Biomolecules. 2024 Sep 3;14(9):1111. doi: 10.3390/biom14091111 (PMC11430817; doi:10.3390/biom14091111)
Supplement: Supplementary file 1 [file biomolecules-14-01111-s001.zip › biomolecules-3152101-suppl file S1.pdf]

Fig. 3

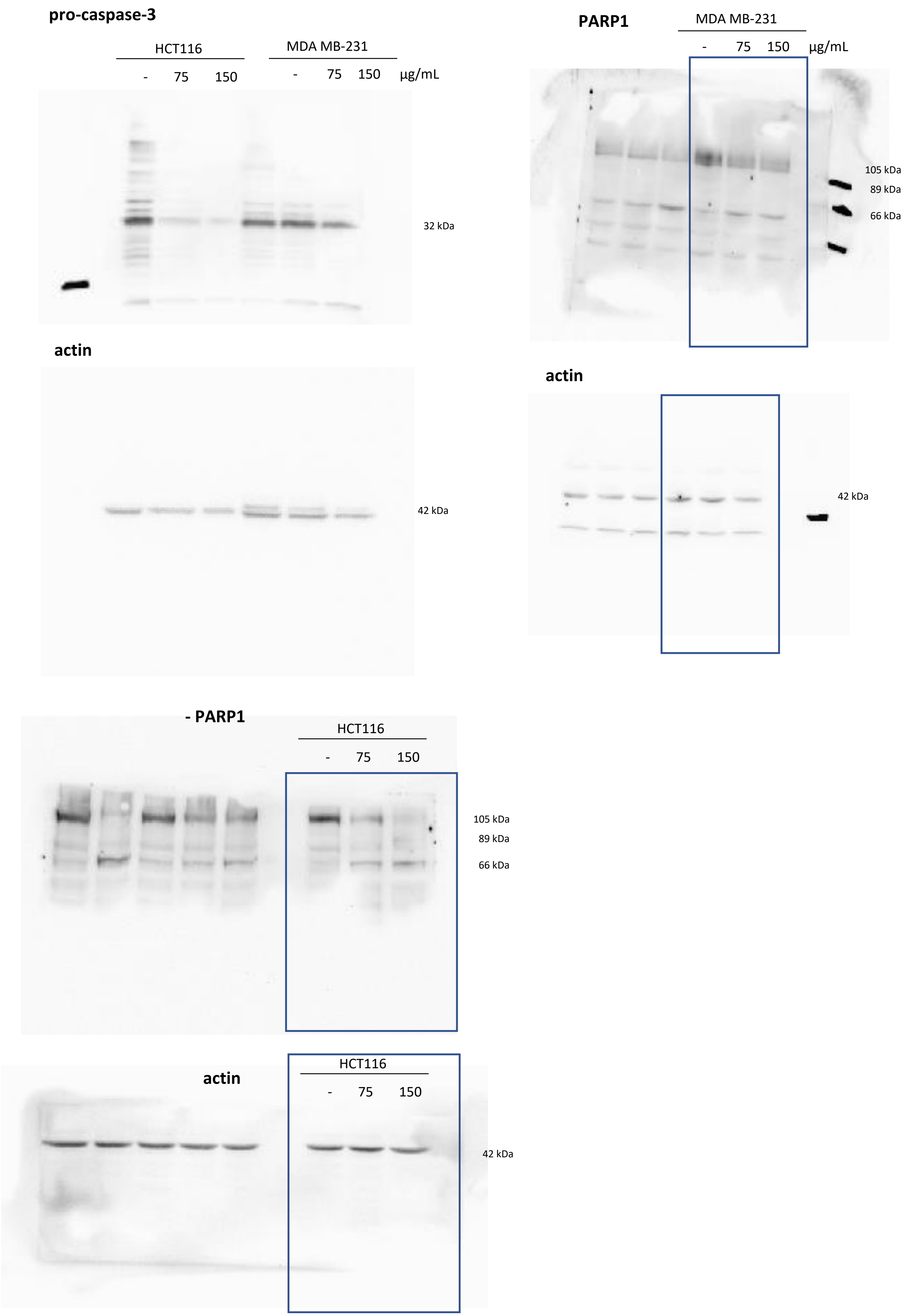

pro-caspase-3
HCT116
MDA MB-231
- 75 150 - 75 150 µg/mL
32 kDa
actin
42 kDa
PARP1
MDA MB-231
- 75 150 µg/mL
105 kDa
89 kDa
66 kDa
actin
42 kDa
- PARP1
HCT116
- 75 150
105 kDa
89 kDa
66 kDa
actin
HCT116
- 75 150
42 kDa

Fig. 4

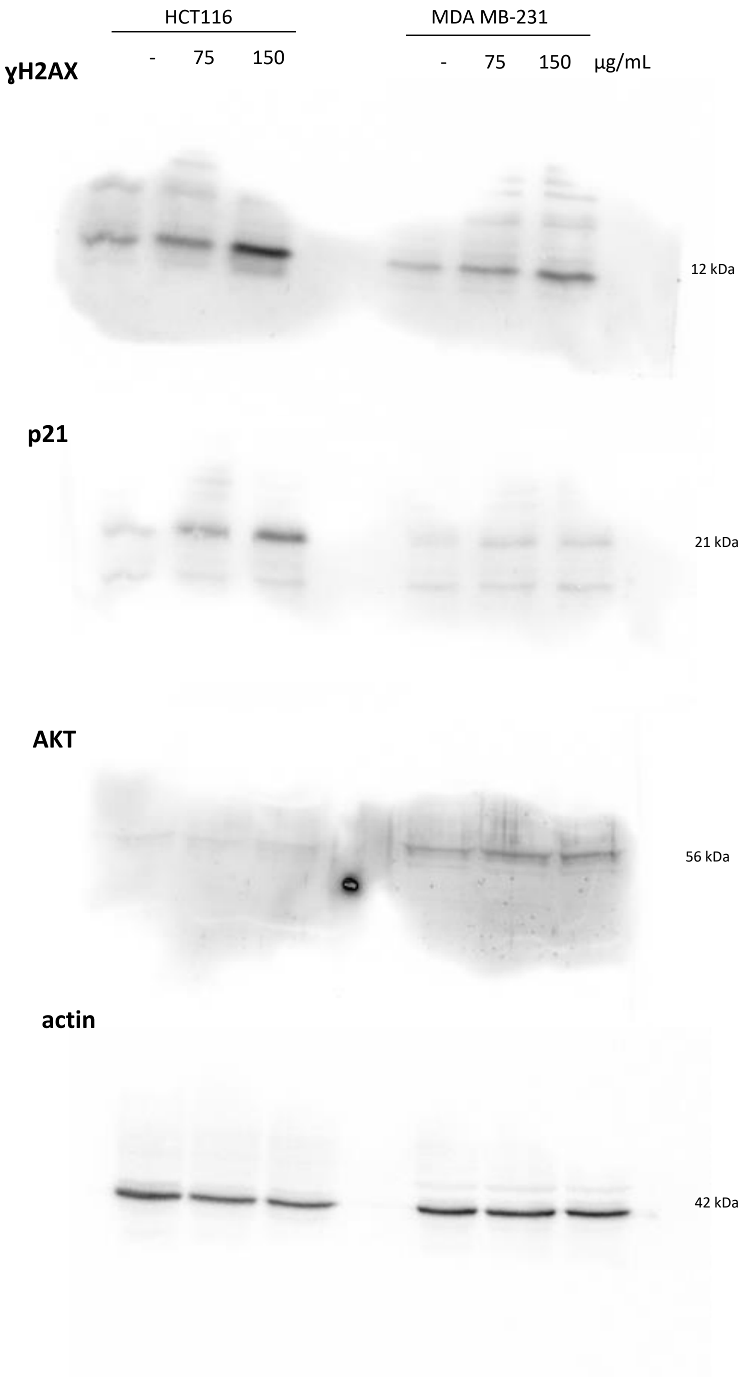
HCT116
MDA MB-231
- 75 150
- 75 150 µg/mL
ɣH2AX
12 kDa
p21
21 kDa
AKT
56 kDa
actin
42 kDa

Fig. 4

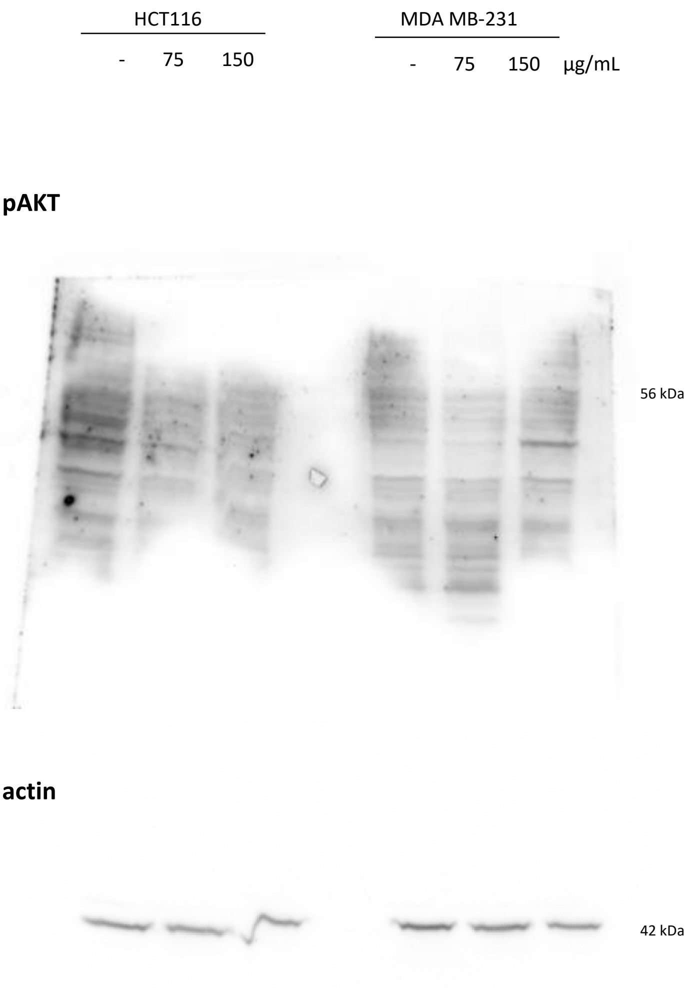

Fig. 6

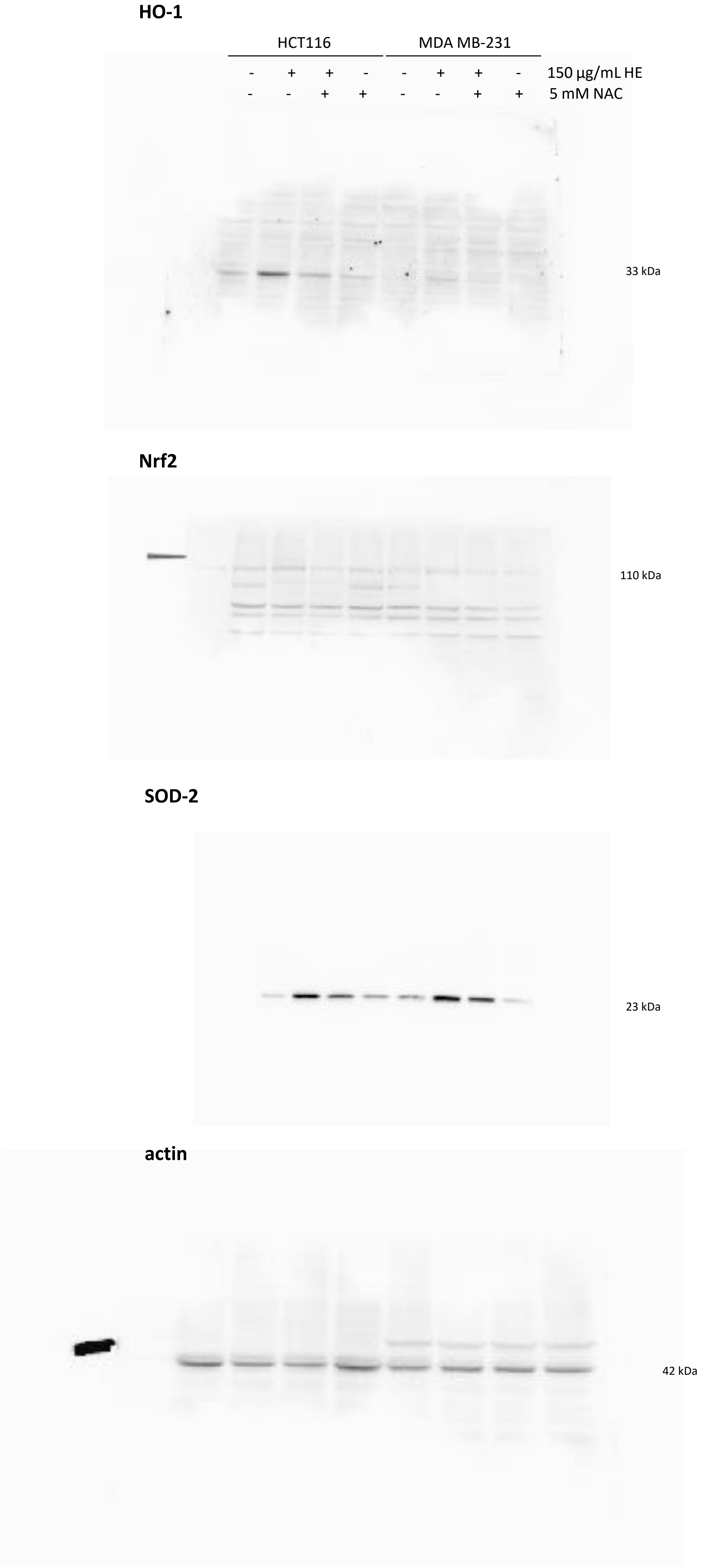
HO-1
HCT116
MDA MB-231
- + + - - + + - 150 µg/mL HE
- - + + - - + + 5 mM NAC
33 kDa
Nrf2
110 kDa
SOD-2
23 kDa
actin
42 kDa

Fig. 8

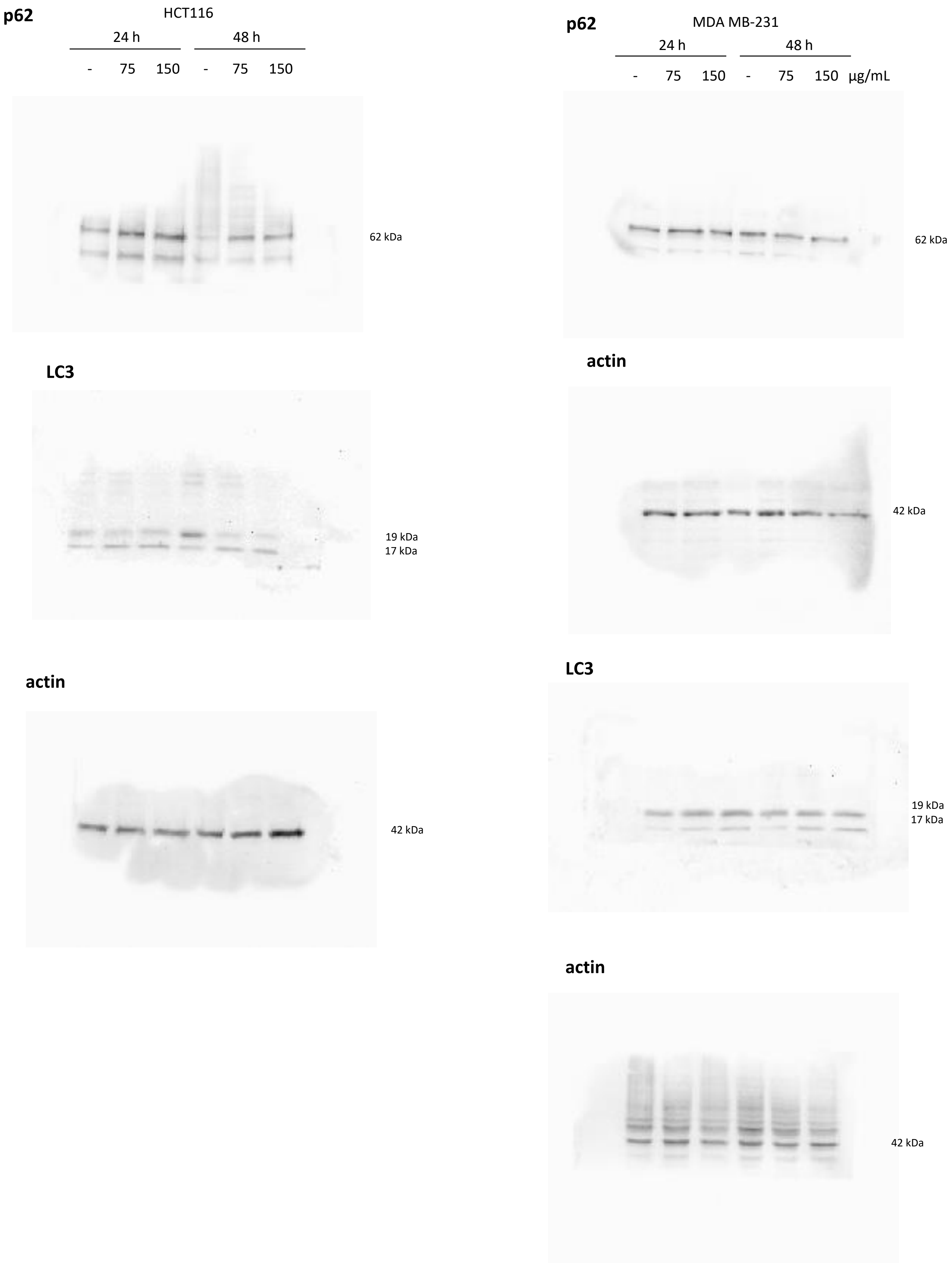
p62
HCT116
24 h
48 h
- 75 150 - 75 150
62 kDa
LC3
19 kDa
17 kDa
actin
42 kDa
p62
MDA MB-231
24 h
48 h
- 75 150 - 75 150 µg/mL
62 kDa
actin
42 kDa
LC3
19 kDa
17 kDa
actin
42 kDa

Fig. 9

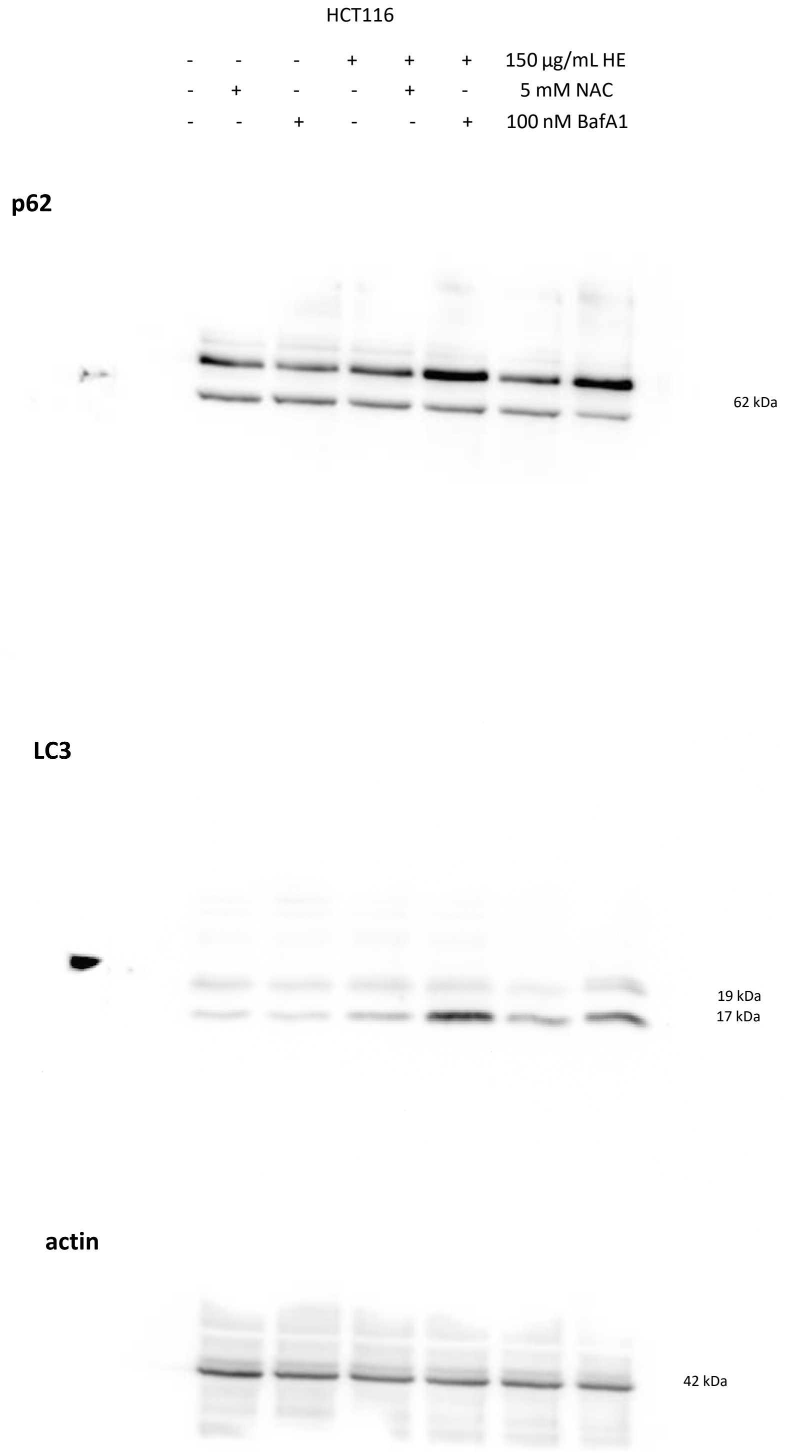
HCT116
- - - + + + 150 µg/mL HE
- + - - + - 5 mM NAC
- - + - - + 100 nM BafA1
p62
62 kDa
LC3
19 kDa
17 kDa
actin
42 kDa

Fig. 9

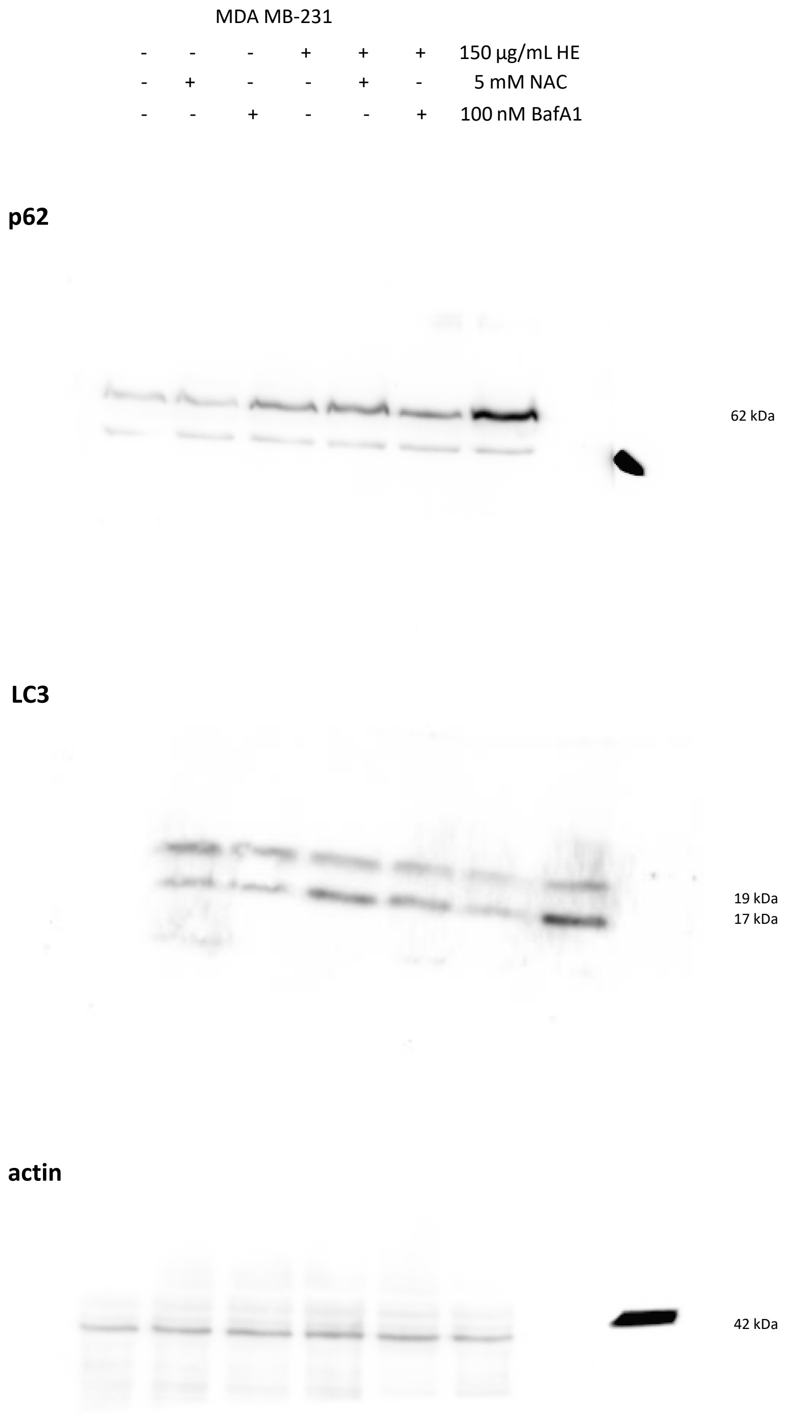
MDA MB-231
- - - + + + 150 µg/mL HE
- + - - + - 5 mM NAC
- - + - - + 100 nM BafA1
p62
62 kDa
LC3
19 kDa
17 kDa
actin
42 kDa

Fig. 10

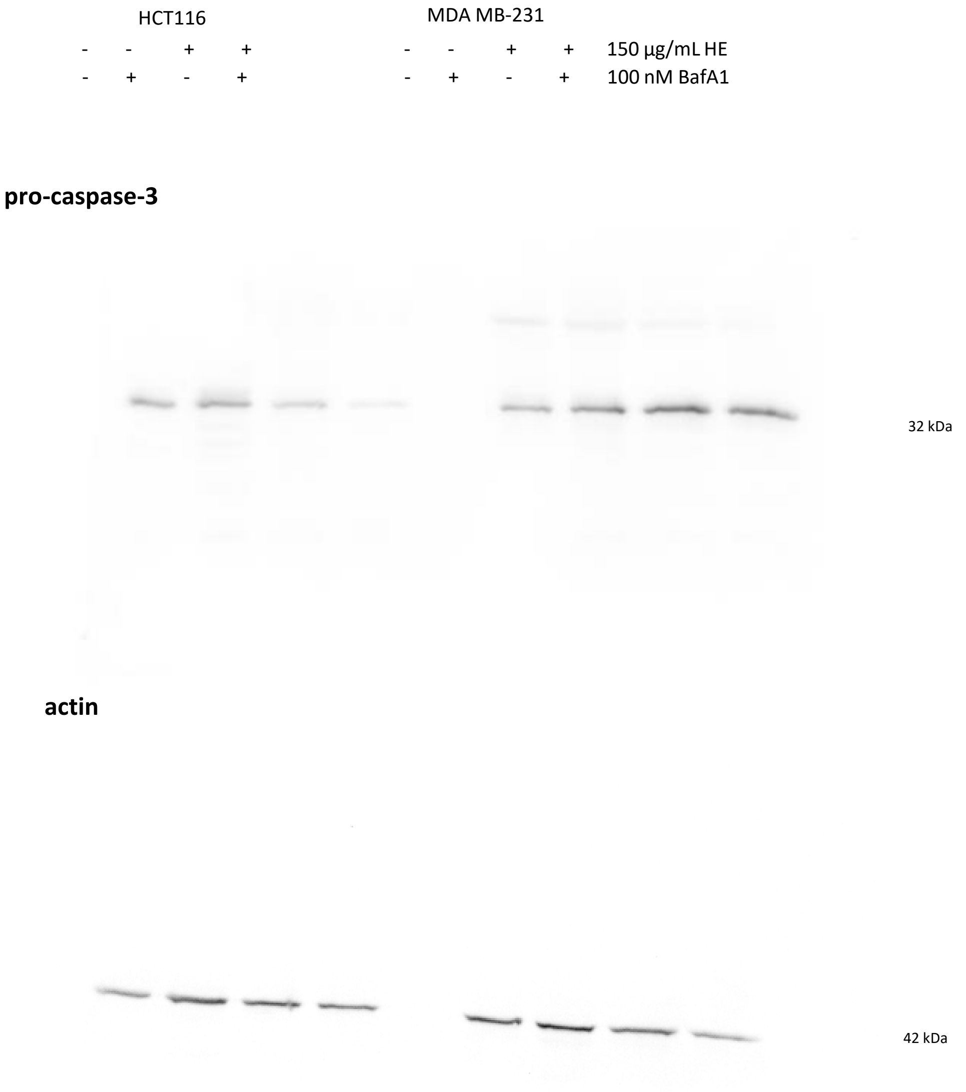
HCT116
MDA MB-231
- - + + - - + + 150 µg/mL HE
- + - + - + - + 100 nM BafA1
pro-caspase-3
32 kDa
actin
42 kDa
